# Enucleation versus hepatectomy for hepatic hemangiomas: A meta-analysis

**DOI:** 10.3389/fsurg.2022.960768

**Published:** 2022-07-28

**Authors:** Bin Jiang, Zheng-Chao Shen, Xiao-San Fang, Xiao-Ming Wang

**Affiliations:** Department of Hepato-Biliary-Pancreatic Surgery, The First Affiliated Hospital of Wannan Medical College (Yijishan Hospital), Wuhu, China

**Keywords:** enucleation, hepatectomy, hepatic hemangiomas, meta-analysis, treatment

## Abstract

**Objective:**

To compare the safety and efficacy of enucleation and hepatectomy for the treatment of hepatic hemangioma (HH).

**Methods:**

A systematic literature search was conducted to identify studies evaluating enucleation versus hepatectomy for HH starting from the time of database creation to February 2022. Extraction of the data used in this study was done from the literature. The differences between the two surgical approaches were evaluated by comparing and analyzing the relevant data by means of meta-analysis.

**Results:**

A total of 1,384 patients (726 underwent enucleation, and 658 with hepatectomy) were included in our meta-analysis from 12 studies. Enucleations were associated with favorable outcomes in terms of operation time [mean difference (MD): −39.76, 95% confidence interval (CI): −46.23, −33.30], blood loss (MD: −300.42, 95% CI: −385.64, −215.19), length of hospital stay (MD: −2.33, 95% CI: −3.22, −1.44), and postoperative complications (OR: 0.57, 95% CI: 0.44–0.74). There were no differences between the groups in terms of patients needing transfusion (OR: 0.85, 95% CI: 0.50, 1.42), inflow occlusion time (MD: 1.72, 95% CI: −0.27, 3.71), and 30-day postoperative mortality (OR: 0.23, 95% CI: 0.02–2.17).

**Conclusion:**

Compared with hepatectomy, enucleation is found to be effective at reducing postoperative complications, blood loss, and operation time and shortening the length of hospital stay. Enucleation is similar to hepatectomy in terms of inflow occlusion time, 30-day postoperative mortality, and patients needing transfusing to hepatectomy.

## Introduction

Hepatic hemangioma is the most common benign tumor of the liver, with an incidence of about 0.4%–20% in the population and usually with high incidence in women aged between 30 and 50 years (1, 2). The most modal pathological type is the cavernous hemangioma (2). There may be no obvious symptoms when the hemangioma is small in diameter. When the hemangioma is large in diameter, gastrointestinal symptoms (such as nausea and vomiting) may occur, and in severe cases, jaundice and ascites may develop (3). At present, follow-up observation is the principal method of treatment for hepatic hemangiomas, especially for asymptomatic patients. Hepatic hemangiomas grow slowly and generally do not require any treatment, and only a small number of patients require surgery (4, 5). There are various treatment methods such as hemangioma enucleation, hepatectomy, hepatic artery interventional embolization, radiofrequency ablation, and others (6, 7). When there are surgical indications such as large tumors, liver resection or hemangioma enucleation is the first choice of treatment (8). Hepatectomy is the traditional surgery for the treatment of hepatic hemangioma, but this surgery may be more traumatic than others because of the excessive removal of normal liver tissues, inaccurate treatment of liver sections, and a large amount of blood leakage. Hemangioma resection can make use of the well-defined fibrous membrane formed by the hepatic hemangioma compressing the normal liver tissue to bluntly peel away the hemangioma, preserving as much normal liver tissue as possible and reducing surgical trauma and hepatobiliary injury.

A series of previous studies have evaluated the surgical outcomes of resection and hepatectomy, but their conclusions are conflicting in nature (9, 10). Against this background, we conducted the present meta-analysis to compare the efficacy of liver resection with that of enucleation for hepatic hemangiomas.

## Methods

Two authors (Xiao-San Fang and Bin Jiang) searched the literature independently. The language of the literature search was restricted, and only English-written articles were included in our meta-analysis. All clinical studies, both prospective and retrospective, that compared enucleation with hepatectomy for HH were included in this meta-analysis. Case reports, reviews, and animal studies were excluded.

We systematically searched PubMed, the Cochrane Collaboration Central Register, Cochrane Systematic Reviews, Web of Science, and Embase until the end of February 2022, along with the references of all articles, which were retrieved in full text. Our search included the key words “laparoscopy,” “laparoscopic,” “minimally invasive,” “open liver resection,” “liver resection,” “hepatectomy,” “Enucleation,” “hepatic hemangiomas,” and “liver hemangiomas.”

Two investigators (Zheng-Chao Shen and Xiao-Ming Wang) independently reviewed the full text of the included studies and extracted information such as patient characteristics, inclusion criteria, and short-term outcomes (operative time, operative blood loss, blood transfusion requirement, portal vein inflow occlusion time, 30-day mortality, length of hospital stay, and postoperative complications).

Discrepancies were resolved by conducting a joint review of the full text to reach a consensus with all authors during data collection, synthesis, and analysis. The quality of all the included studies was assessed independently by the same reviewers with the Methodological Index for Non-Randomized Studies (11) since all the studies included in our meta-analysis were non-randomized. Our meta-analysis was performed using Review Manager Version 5.3.5 software (The Nordic Cochrane Centre, The Cochrane Collaboration, Copenhagen, 2014). First, heterogeneity across all studies was evaluated using the *χ*^2^ test (Chi-square test), and a *P*-value <0.05 was considered statistically significant. If research studies had homogeneity, the fixed effects model was used for calculating combined statistics, and in case of heterogeneity, random-effects analysis was used. For categorical variables, treatment effects were expressed as the odds ratio (OR) with a corresponding 95% CI, and for continuous variables, treatment effects were expressed as the mean difference (MD). Confidence intervals were set at 95%. Funnel plots, Egger test, and Begg test were used to evaluate potential publication bias.

## Results

**Figure 1:**
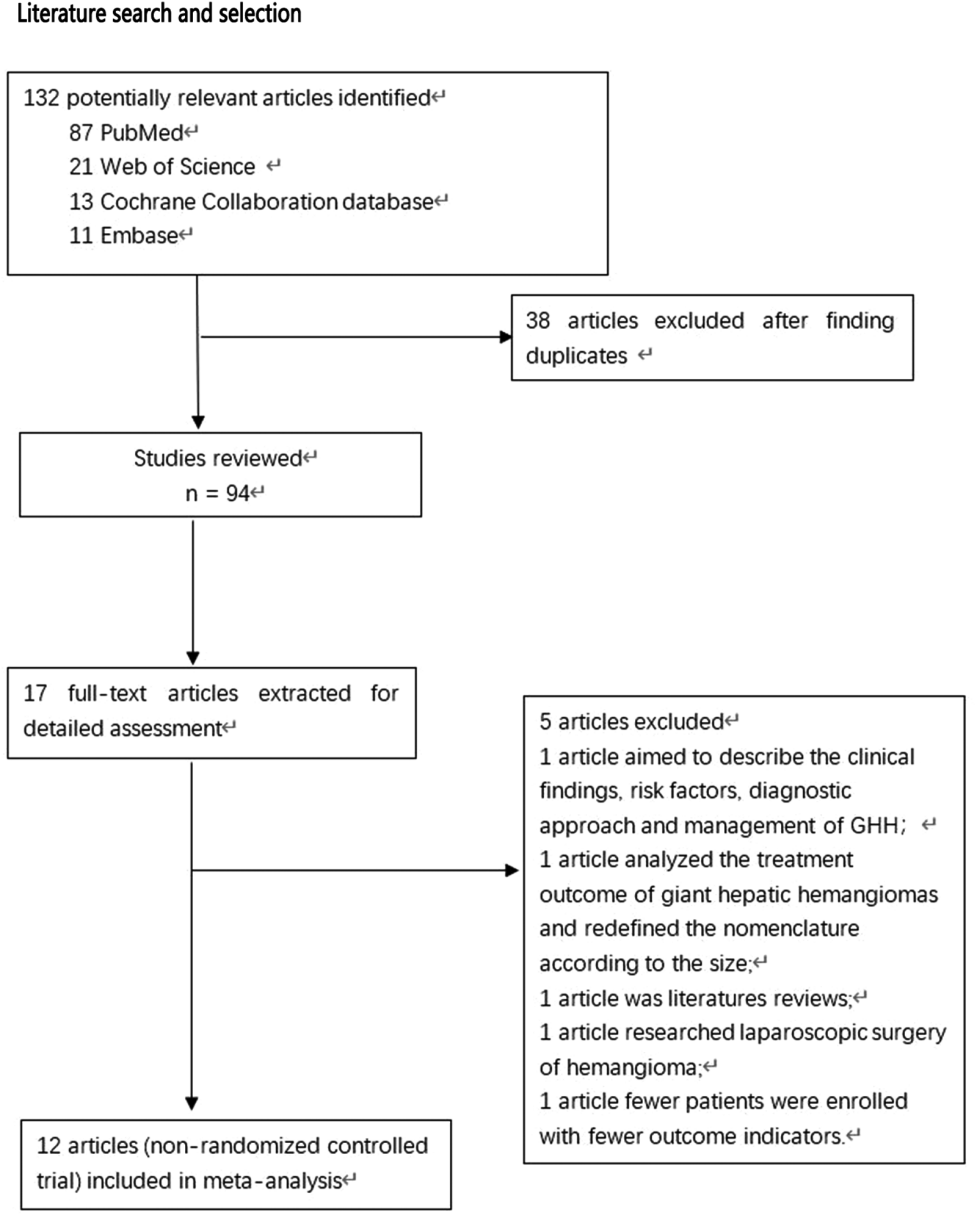
PRISMA flowchart.

During the preliminary search, 132 articles were obtained. After reading the titles and abstracts and full texts and removing duplicate documents, a final number of 12 articles (8, 12–22) were included in our study, which involved 1,384 patients (Figure 1). Among them, 726 underwent pure hemangiomas enucleation and 658 were operated through hepatectomy. The methodological characteristics of the included studies (Table 1), the characteristics of the enrolled patients (Table 2), and the outcomes of the operations among the mentioned groups (Table 3) are listed in the tables. The results of the overall meta-analysis are outlined in Table 4.

**Table 1:** Characteristics of studies included in this meta-analysis of enucleation vs. hepatectomy for hepatic hemangiomas.

Author	Country	Type of study	MINORS
Kuo et al. (16)	USA	NRCT	20
Gedaly et al. (12)	USA	NRCT	21
Lerner et al. (17)	USA	NRCT	22
Hamaloglu et al. (14)	Turkey	NRCT	20
Singh et al. (20)	India	NRCT	19
Giuliante et al. (13)	Italy	NRCT	23
Qiu et al. (18)	China	NRCT	23
Zhang et al. (22)	China	NRCT	20
Wu et al. (21)	China	NRCT	20
Abdel et al. (8)	Egypt	NRCT	22
Ju et al. (15)	China	NRCT	24
Rajakannu et al. (19)	France	NRCT	20

NRCT, non-randomized controlled trials.

**Table 2:** Characteristics of patients with hepatic hemangiomas who underwent surgery (enucleation vs. hepatectomy).

Study	Intervention	Patients (*n*)	Sex (M/F)	Age (years)	Tumor size (cm)
Kuo et al. (16)	Enucleation	10	NA	NA	7.6 ± 1.3
	Hepatectomy	10	NA	NA	8.4 ± 1.2
Gedaly et al. (12)	Enucleation	23	NA	47.6 ± 13.4	6 ± 3.7
	Hepatectomy	5	NA	47.2 ± 7.2	8.6 ± 5.4
Lerner et al. (17)	Enucleation	27	1/27	45 ± 9.2	10.1 ± 5.3
	Hepatectomy	25	5/20	51 ± 10.5	11.6 ± 4.3
Hamaloglu et al. (14)	Enucleation	10	NA	41.5 ± 6.7	7.8 ± 0.7
	Hepatectomy	12	NA	46.0 ± 6.8	8.1 ± 0.8
Singh et al. (20)	Enucleation	9	NA	NA	8.9 ± 3.3
	Hepatectomy	12	NA	NA	10 ± 6.2
Giuliante et al. (13)	Enucleation	12	NA	NA	12.3 ± 12.9
	Hepatectomy	28	NA	NA	11.8 ± 9.2
Qiu et al. (18)	Enucleation	386	224/162	45 ± 8.33	6.7 ± 6.9
	Hepatectomy	344	205/139	46 ± 7.83	6.9 ± 2.3
Zhang et al. (22)	Enucleation	32	8/24	42.1 ± 9.5	13.1 ± 3.6
	Hepatectomy	11	1/10	45.8 ± 9.6	15.2 ± 6.1
Zhang et al. (22)*	Enucleation	6	1/5	44.5 ± 6.7	14.8 ± 5.4
	Hepatectomy	24	12/12	50.2 ± 8.2	12.8 ± 3.8
Wu et al. (21)	Enucleation	31	7/24	45.4 ± 8.9	13.9 ± 3.1
	Hepatectomy	22	4/18	47.6 ± 11.7	12.3 ± 5.5
Abdel et al. (8)	Enucleation	92	38/54	43 ± 6.67	11.5 ± 4.33
	Hepatectomy	52	18/34	46 ± 10.25	10 ± 5
Ju et al. (15)	Enucleation	66	50/16	47.6 ± 9.4	10.2 ± 2.9
	Hepatectomy	66	48/18	47.0 ± 9.0	10.1 ± 3.5
Rajakannu et al. (19)	Enucleation	22	4/18	50.75 ± 9.75	16.31 ± 5.68
	Hepatectomy	32	4/28	47 ± 0.75	14 ± 6.75

NA, not available.

* Represents information from another group of patients in the same original study (Zhang et al. (22)).

**Table 3:** The outcomes following enucleation or hepatectomy in patients with hepatic hemangiomas.

Study	Intervention	Operation time (minutes)	Blood loss (ml)	Patients needing transfusion (*n*)	Inflow occlusion time (minutes)	Length of stay (days)	30-day Postoperative mortality (*n*)	Postoperative complications (*n*)
Kuo et al. (16)	Enucleation	132 ± 18	400 ± 129	1	NA	9.5 ± 1.2	0	0
	Hepatectomy	144 ± 12	742 ± 116	3	NA	9.1 ± 1.8	0	2
Gedaly et al. (12)	Enucleation	204 ± 72	923 ± 1033	NA	14 ± 18	8.2 ± 3.9	0	8
	Hepatectomy	258 ± 90	2080 ± 1139	NA	19 ± 20	9.4 ± 2.2	0	4
Lerner et al. (17)	Enucleation	174 ± 72	NA	4	23 ± 12	6.9 ± 2.2	0	3
	Hepatectomy	198 ± 65	NA	7	15 ± 9	8.7 ± 4.1	0	11
Hamaloglu et al. (14)	Enucleation	110 ± 27	150 ± 189	NA	NA	5.0 ± 1.0	0	1
	Hepatectomy	190 ± 95	375 ± 339	NA	NA	7.0 ± 3.9	0	2
Singh et al. (20)	Enucleation	175 ± 35	400 ± 116	NA	NA	5.8 ± 3.54	0	0
	Hepatectomy	223 ± 78	1329 ± 1485	NA	NA	10.95 ± 7.83	0	5
Giuliante et al. (13)	Enucleation	323 ± 138	NA	2	79 ± 50	7.75 ± 4.91	0	1
	Hepatectomy	260 ± 140	NA	4	48 ± 26	10.3 ± 8	0	3
Qiu et al. (18)	Enucleation	150 ± 40.83	400 ± 75	NA	NA	5.7 ± 0.83	0	68
	Hepatectomy	240 ± 58.33	860 ± 158	NA	NA	8.6 ± 2.17	0	97
Zhang et al. (22)	Enucleation	201.1 ± 63.5	400 ± 50	9	13.5 ± 5.2	12.2 ± 3.8	0	12
	Hepatectomy	237.1 ± 49.0	550 ± 392.64	4	17.5 ± 8.5	12.0 ± 3.3	0	4
Zhang et al. (22)*	Enucleation	221.0 ± 46.2	362.5 ± 333.62	2	14.0 ± 2.2	10.8 ± 1.9	0	2
	Hepatectomy	215.8 ± 64.7	575 ± 375	4	11.5 ± 3.2	12.0 ± 2.7	0	5
Wu et al. (21)	Enucleation	102 ± 24	350.9 ± 89.8	NA	30.4 ± 0.5	9.6 ± 4.2	0	1
	Hepatectomy	174 ± 54	988 ± 91.7	NA	32.8 ± 3.5	14.7 ± 3.7	0	5
Abdel et al. (8)	Enucleation	138 ± 53.3	424 ± 1658.33	35	NA	5 ± 4.66	0	21
	Hepatectomy	165 ± 67.5	350 ± 1350	49	NA	6 ± 5.25	1	11
Ju et al. (15)	Enucleation	156 ± 20	200 ± 68.75	22	NA	9.5 ± 2.6	0	8
	Hepatectomy	195 ± 24.75	220 ± 65	22	NA	9.0 ± 1.9	0	11
Rajakannu et al. (19)	Enucleation	296.25 ± 116.25	3050 ± 2800	NA	53.25 ± 26.75	12.75 ± 6.25	0	9
	Hepatectomy	203 ± 95.75	400 ± 1750	NA	39 ± 29.5	10 ± 4.08	2	12

NA, not available.

* Represents information from another group of patients in the same original study (Zhang et al. (22)).

**Table 4:** Results of a meta-analysis comparing enucleation and hepatectomy for hepatic hemangioma.

Outcome of interest	No. of studies	No. of patients		OR/MD	95% CI	*P*-value	Heterogeneity	
		Enucleation	Hepatectomy				*P*-value*	*I*^2^ (%)
Sex (M)	7	662	576	0.99	(0.78,1.26)	0.93	0.41	3
Age (years)	9	695	593	−1.16	(−2.10,−0.23)	0.01	0.10	38
Tumor size (cm)	12	726	643	−0.16	(−0.53,0.22)	0.41	0.32	12
Operation time (minutes)	8	296	244	−39.76	(−46.23,−33.30)	<0.00001	0.05	47
Blood loss (ml)	5	90	74	−300.42	(−385.64,−215.19)	<0.00001	0.19	33
Patients needing transfusion (*n*)	6	153	164	0.85	(0.50,1.42)	0.53	0.66	0
Inflow occlusion time (minutes)	6	83	72	1.72	(−0.27,3.71)	0.09	0.05	63
Length of stay (days)	7	118	128	−2.33	(−3.22,−1.44)	<0.00001	0.11	42
30-day postoperative mortality (*n*)	12	726	643	0.23	(0.02,2.17)	0.20	0.87	0
Postoperative complications (*n*)	12	726	643	0.57	(0.44,0.74)	<0.00001	0.22	22

OR, odds ratio; MD, mean difference.

* Represents information from another group of patients in the same original study (Zhang et al. (22)).

### Operative outcomes

**Figure 2:**
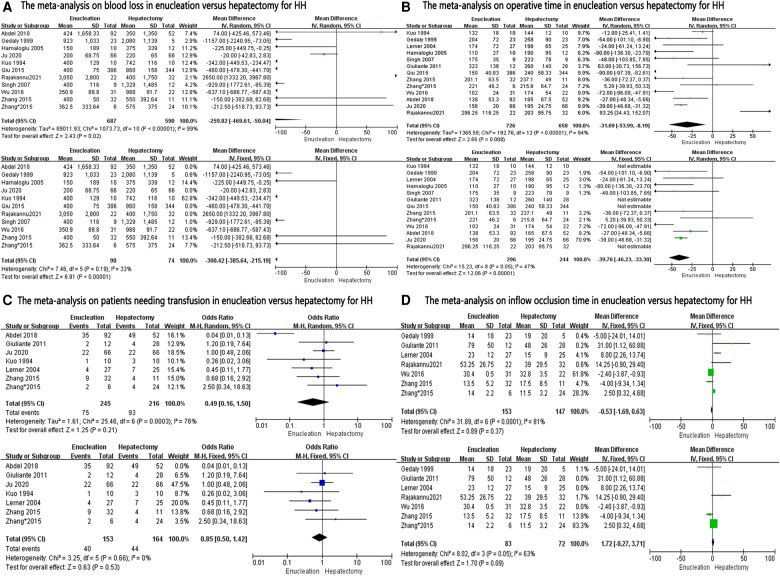
The forest plots for operative outcomes in enucleation versus hepatectomy for HH.

A data analysis of the ten reports included in our study showed that there was no significant difference in blood loss between the two groups (MD: −259.82, 95% CI: −2–469.61, −50.04; *P* = 0.02), with significant heterogeneity between studies (*I*^2^ = 99%) (Figure 2A). Because of this high heterogeneity (*I*^2 ^= 99%, *P* < 0.00001), the Galbraith plot test was used to detect the potential sources of heterogeneity (Supplementary Figure S1). According to the results of the detection, we excluded some studies that might have been the sources of heterogeneity. Blood loss was found to be significantly lower in the enucleation group than in the hepatectomy group (MD, −330.42; 95% CI, −385.64, −215.19; *P* < 0.00001), with significant lower heterogeneity between the studies (*I*^2^ = 33%, *P* = 0.19) (Figure 2A).

Twelve studies provided data on operation time. Analysis showed that operation time was significantly less in the enucleation group (MD: −31.09, 95% CI: −53.99, −8.19; *P* = 0.008), with significant heterogeneity between the studies (*I*^2^ = 94%, *P* < 0.00001) (Figure 2B). After excluding some studies that might have been sources of heterogeneity, operation time was also found to be less in the enucleation group than in the hepatectomy group. (MD: −39.76, 95% CI: −46.23, −33.30; *P* < 0.00001, *I*^2^ = 47%, *P* = 0.05).

For patients needing transfusion, inflow occlusion time was mentioned in six studies, and there was no significant difference between the two groups (OR: 0.49, 95% CI: 0.16, 1.50; *P* = 0.21, *I*^2 ^= 76%, *P* = 0.0003; and MD: −0.53, 95% CI: −1.69, 0.63, *I*^2^ = 81%, *P* < 0.0001, respectively). Even after excluding some studies that might have been sources of heterogeneity, no significant difference was found between the two groups (OR: 0.85, 95% CI: 0.50, 1.42; *P* = 0.53, *I*^2 ^= 0%, *P* = 0.66; and MD: 1.72, 95% CI: −0.27, 3.71, *P* = 0.09, *I*^2 ^= 63%, *P* = 0.05, respectively) (Figures 2C,D).

### Postoperative outcomes

**Figure 3:**
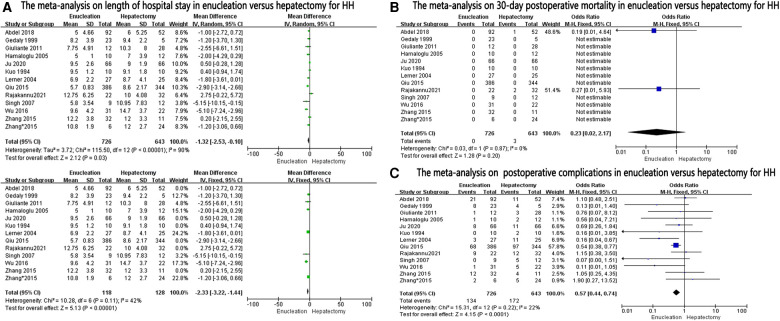
The forest plots for postoperative outcomes in enucleation versus hepatectomy for HH.

Length of hospital stay was reported in 12 studies and was found to be significantly lower in the enucleation group than in the hepatectomy group (MD, −1.32; 95% CI, −2.53, −0.10; *P* = 0.03), with significant heterogeneity between the studies (*I*^2^ = 90%, *P* < 0.0001). After excluding some studies on the ground that they might be sources of heterogeneity, it was found that the length of hospital stay in the enucleation group was still higher than that in the hepatectomy group (MD: −2.33, 95% CI: −3.22, −1.44, *P* < 0.0001, *I*^2^ = 42%, *P* = 0.11) (Figure 3A).

The thirty-day postoperative mortality rate was calculated in 12 studies, and no significant difference was found between the two groups (OR: 0.23, 95% CI: 0.02–2.17; =0.20, *I*^2 ^= 0%, *P* = 0.87) (Figure 3B).

Postoperative complications in the enucleation group were significantly lower than those in the hepatectomy group (OR: 0.57, 95% CI: 0.44–0.74, *P* < 0.0001, *I*^2 ^= 22%, *P* = 0.22), provided in 12 studies (Figure 3C).

## Discussion

With the rapid development of minimally invasive techniques, interventional techniques such as hepatic artery embolization and radiofrequency ablation are commonly used for the treatment of hepatic hemangioma, but surgery is still the most effective treatment for hepatic hemangioma, including hepatectomy and enucleation (18, 23–27). However, the choice between the two procedures still depends on the surgeon’s personal preference. In the year 1898, Hermann Pfannenstiel first reported hepatectomy for treating hepatic hemangiomas, and for some time, this method remained the only effective treatment (28). A new technique for hemangioma enucleation was discovered by Alper et al. 90 years later in 1988, that after incising the liver capsule, the dissection was performed in the cleavage plane between the capsule of the hemangioma and surrounding liver tissue (29). During liver resection, the extent of resection should be determined according to the location, the number of hemangiomas, and the adjoining relationship between the tumor and the surrounding tissue. If the tumor is huge and its anatomical location is complex, massive bleeding will easily result and endanger the life and health of the patient (30). Most hepatic hemangiomas show expansile growth, compressing the surrounding liver tissue, blood vessels, and bile ducts, forming a fibrous membrane visible to the naked eye. This special capsule is the anatomical basis of hemangioma enucleation (15). Enucleating the hemangioma along the edge of the capsule can ensure the complete removal of the hemangioma, while preserving a maximum amount of the normal liver tissue and reducing intraoperative hepatobiliary injury (15, 31). Although enucleating is theoretically more effective and safer than traditional liver resection, clinical scholars disagree on this. Traditional liver resection is advocated by some authors (32, 33). For example in cases of large and deep hemangiomas proximity to vascular structures, typical liver resection is a safe operation with lower mortality and blood loss. Others, however, advocate enucleation (29, 34), believing that this technique avoids the need to resect normal liver parenchyma and minimizes damage to blood vessels and bile ducts. Some scholars feel that both hemangioma enucleation and liver resection have similar curative effects, and therefore, both can completely eliminate hemangioma and improve the quality of life of patients (14). Our meta-analysis aims to further evaluate the perioperative safety, practicality, and effectiveness of enucleation relative to hepatectomy for hepatic hemangiomas.

In terms of blood loss, the results of our analysis showed a lower amount in the enucleation group than in the hepatectomy group, both before and after the exclusion of heterogeneity, similar to previous reports (9). The main causes of hemorrhage in liver surgery are summarized as follows: (1) Poor exposure to the operative field and accidental injury to large blood vessels. The huge tumor blocked the surgical field of view, and the surgical space was limited, so it was easy to puncture or cut the large blood vessels. At this time, the tumor could not be removed immediately for a while, which caused bleeding that was difficult to control. At this time, the surgical field is even less clear because of bleeding, and it is easy to damage the large blood vessels that are located at the bottom of the tumor, resulting in more serious hemorrhage. This is often seen when the tumor is located in the second hepatic hilar, the deeper part of the middle liver, the paravalvular vein, and the caudal lobe. (2) Excessive traction on the liver ruptures large blood vessels. During resection of the right half of the liver or a huge tumor in the right posterior lobe, the short hepatic vein, right hepatic vein, and right adrenal vein are easily torn during the process of turning the liver to the left after freeing the ligament, and massive bleeding occurs. The narrow surgical field of view and the difficulty of hemostatic operation make them vulnerable to fatal danger. (3) Severe adhesions in the operative field make the operation difficult. In cases of tumor on top of the diaphragm or repeated liver surgery, when separating the adhesions, it is easy to cause the liver tissue to break or the tumor to rupture, thus causing massive bleeding. At this time, if the hemorrhage is stopped by blind clamping, it will lead to the tumor rupture surface getting larger and larger, making it difficult to control the bleeding. This situation mostly occurs in patients with hemangioma located at the top of the hepatic diaphragm, the bare liver area, and the right posterior lobe. (4) Unfamiliarity with an intrahepatic anatomical structure and error of judgment. Due to the pushing of the huge tumor, the large blood vessels in the liver are displaced and the vascular alignment is changed, resulting in error in judgment for the surgeon, which causes large vessel injury and bleeding. For example, right hepatectomy injures the posterior hepatic vena cava or the left inner lobe, or the left hepatectomy accidentally injures the root of the middle hepatic vein or the root of the left hepatic vein. In contrast, especially in liver segments with deep tumor locations and difficult exposure, hepatectomy requires a greater involvement of the large blood vessels and bile ducts of the liver, whereas hemangioma debulking requires only a separation of the tumor from normal liver tissue along a clear demarcation line, usually with relatively few or small bile ducts or blood vessels between the tumor and normal liver tissue.

This study is not without certain limitations. A large number of randomized controlled studies are needed to validate the comparison of the two surgical methods for hemangiomas in different liver locations, as well as carry out an independent analysis between studies that use minimally invasive surgery both in enucleations and in liver resections. However, due to the insufficient amount of available literature, it is not possible to perform such studies or carry out this analysis for the time being, and therefore, we will continue to follow up and update these aspects of the study in the future.

**Figure 4:**
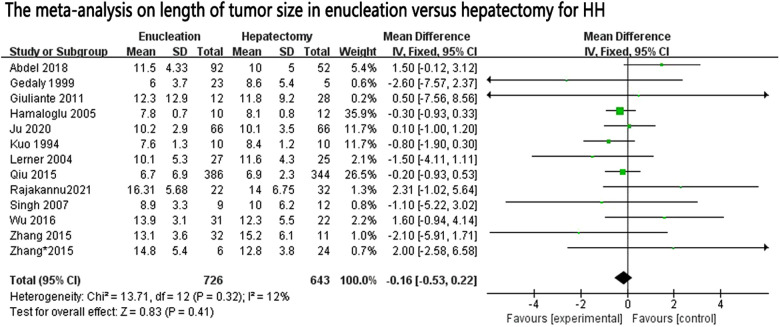
The forest plot for tumor size in enucleation versus hepatectomy for HH.

In our research, it was shown that the operation time was significantly less in the enucleation group than in the hepatectomy group, but with significant heterogeneity between the studies (*I*^2^ = 94%). Although some studies of operative times were excluded according to the Galbraith plot test, heterogeneity still existed, and random effect models were used. To the best of our knowledge, the length of operation time usually has a certain relationship with the difficulty of performing the operation, the smooth conduct of the operation, the size of the tumor, and the technical level of the chief surgeon. We also compared the size of the tumors included in the study, and there was no significant difference in tumor size between the two groups (MD: 0–0.16, 95% CI: −0.53, 0.22; *P* = 0.41, *I*^2 ^= 12%, *P* = 0.32) (Figure 4). As analyzed above, hepatectomy damages blood vessels and bile ducts more frequently, and the surgeon usually spends more time in ligation and hemostasis, and the continuity of surgery is also disrupted (9).

In our research, for patients needing transfusion, there was no significant difference between the two groups in terms of inflow occlusion time (OR: 0.85, 95% CI: 0.50, 1.42; *P* = 0.53, *I*^2 ^= 0%, *P* = 0.66; and MD: 1.72, 95% CI: −0.27, 3.71, *P* = 0.09, *I*^2 ^= 63%, *P* = 0.05, respectively), which contrasts previous studies. Inflow occlusion is a common method to prevent bleeding during liver surgery. Pringle maneuver was first applied by Pringle in the year 1908, and today, hepatobiliary surgeons are quite familiar with it (35). With the development of surgical techniques, different occluding methods have been proposed and applied in the clinic, such as hemihepatic occlusion and hepatic segment vascular occlusion (36). The effective hepatic inflow occlusion method reduces intraoperative bleeding and provides a clear surgical field.

In the present study, there was no statistical difference between the two groups in terms of 30-day postoperative mortality. With the improvement in surgical techniques and the surgeon’s profound understanding of liver anatomy, whether it is hepatectomy or enucleation, the operation methods have gradually become mature and procedural in nature, and sufficient preoperative preparation will reduce the risk of death in the perioperative period (14). With the advances in minimally invasive surgical techniques in particular, almost all laparoscopic techniques have become unrestricted in the field of liver surgery. For the treatment of hepatic hemangiomas, laparoscopic hepatectomy is fundamentally a surgical operation that is not significantly different from hepatectomy for other liver tumors. The safety and efficacy of the laparoscopic hepatectomy technique for the treatment of liver tumors has been confirmed by a considerable number of researchers (37). In contrast, fewer studies have been reported on the treatment of hepatic hemangioma by laparoscopic enucleations. In particular, few controlled studies have been retrieved on the enucleations of hepatic hemangiomas under laparoscopic surgery versus open surgery. Because of the problems pertaining to the operating angle and the operating orientation in laparoscopic surgery, the selection of the correct operation path for the enucleations of hepatic hemangiomas is of great importance for the successful completion of the surgery. Combining our surgical experience and previous literature, we summarize the technical key points of laparoscopic hepatic hemangioma enucleations for the benefit and reference of hepatobiliary surgeons. (1) Selection of the location of the main operating ports under laparoscopy. The apex of the surgical approach should correspond to the main operating ports during surgery. (2) Selection of the starting surgical point. For tumors located at the edge of the liver, it is recommended to select the edge of the liver as the starting point. Previous studies have found that the blood supply of hepatic hemangioma mostly enters from the lower pole of the tumor. After regional blockage of hepatic blood flow, the tumor becomes soft and has a certain boundary with normal liver tissue, so it is relatively easy to find the lower pole envelope of the tumor by first adopting a bottom-up approach and then a peripheral peeling approach to avoid bleeding. (3) Pringle maneuver was used to block hepatic blood flow. Most of the livers of patients with hepatic hemangioma have normal texture and good liver reserve function, so intermittent hepatic portal block has little effect on postoperative liver function. The Pringle maneuver is simple and time-saving, and the tension of the tumor was reduced after blocking, which is easy to clamp and pull, and the blood leakage from the trauma surface is obviously reduced, which is conducive to a clear surgical field.

For postoperative complications, lower incidence after enucleation was revealed by the present analysis. As discussed above, compared with liver resection, the blood vessels and bile ducts involved in hepatic hemangioma resection are smaller, and the loss of normal liver tissue volume is also smaller, which can reduce postoperative bleeding, bile leakage, and reduce the risk of liver failure, to a certain extent.

In terms of the postoperative outcomes of these two procedures, a shorter length of hospital stay after enucleation was presented in the present analysis. In the enucleation group, due to the shortened operation time and the reduction of postoperative complications, the length of hospital stay was naturally shortened.

We have to admit that this study may have some serious limitations, apart from the limitations mentioned above. First of all, the included studies are all non-randomized controlled studies, and some studies in the literature include only a small number of patients and incomplete outcome indicators, so the evidence from these studies is not of the highest quality. Secondly, because the location of the tumours in the included literature may vary from study to study. And the difficulty of surgery may vary for tumours in different locations, thus making this study somewhat heterogeneous. The heterogeneity of the patients included may have influenced the conclusions. Considering the limitations and heterogeneity in our selected studies, the therapeutic efficacy of enucleation will demand large and well-designed prospective studies to be conducted in the future.

In conclusion, the results of this meta-analysis of 12 articles showed that enucleation for hepatic hemangiomas was superior to hepatectomy with regard to operation time, blood loss, postoperative complications, and length of hospital stay, and that in terms of patients needing a transfusion, inflow occlusion time, and 30-day postoperative mortality, the patient profiles were similar for both enucleation and hepatectomy groups.

## Data Availability

The original contributions presented in the study are included in the article/Supplementary Material; further inquiries can be directed to the corresponding author/s.
