# Supplementary figures and images for: Enucleation versus hepatectomy for hepatic hemangiomas: A meta-analysis
Source: Front Surg. 2022 Jul 28;9:960768. doi: 10.3389/fsurg.2022.960768 (PMC9366102; doi:10.3389/fsurg.2022.960768)

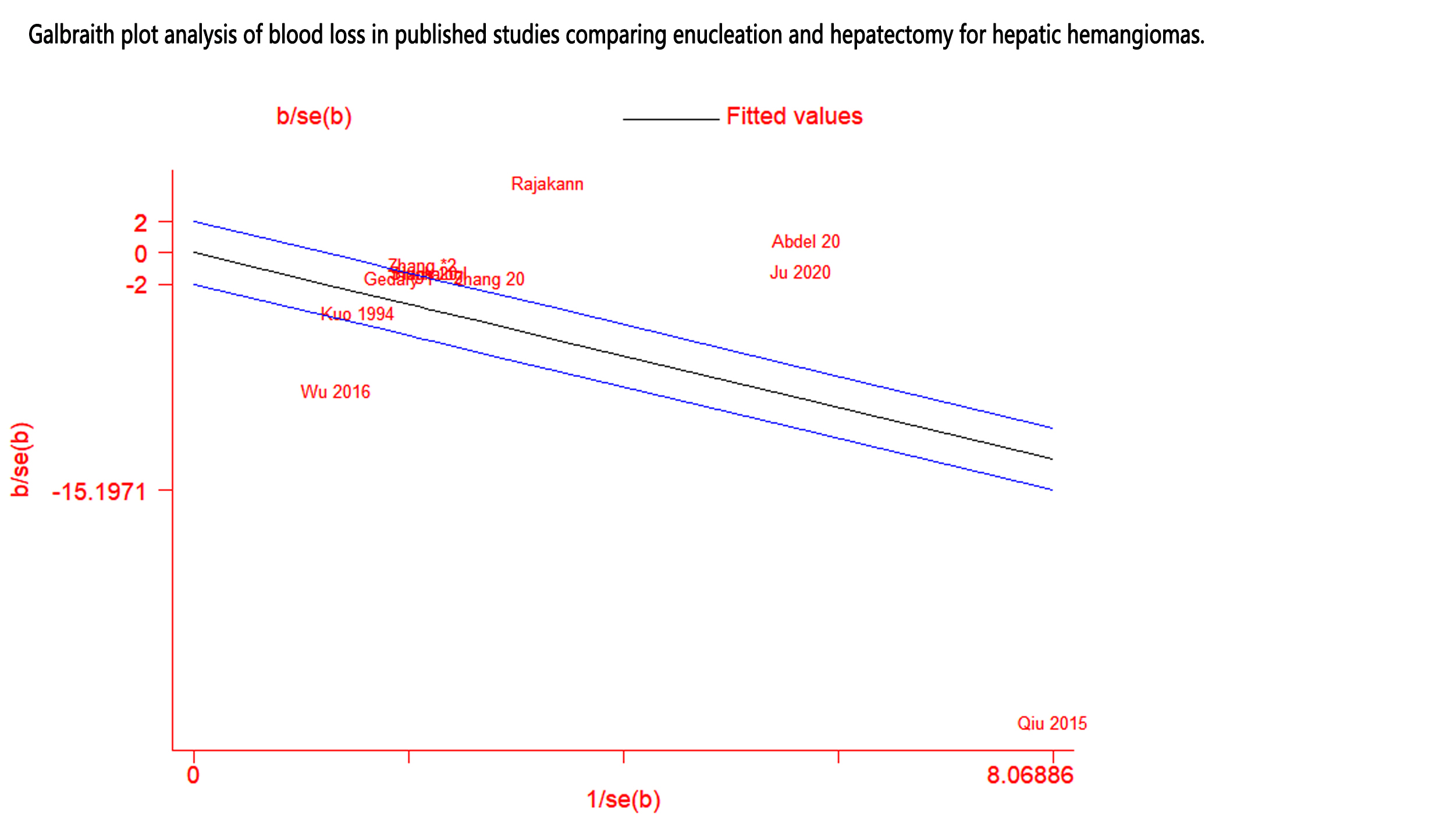

Supplement: Supplementary file 1 [file Image_1_v1.jpeg]
